# Deciphering lung adenocarcinoma evolution: Integrative single‐cell genomics identifies the prognostic lung progression associated signature

**DOI:** 10.1111/jcmm.18408

**Published:** 2024-06-04

**Authors:** Pengpeng Zhang, Zijun Yang, Zuo Liu, Ge Zhang, Lianmin Zhang, Zhenfa Zhang, Jun Fan

**Affiliations:** ^1^ Department of Lung Cancer, Tianjin Lung Cancer Center, National Clinical Research Center for Cancer, Key Laboratory of Cancer Prevention and Therapy, Tianjin's Clinical Research Center for Cancer Tianjin Medical University Cancer Institute and Hospital Tianjin China; ^2^ Department of Cardiology The First Affiliated Hospital of Zhengzhou University Zhengzhou China; ^3^ Department of Thoracic Surgery The First Affiliated Hospital of Nanjing Medical University Nanjing China

**Keywords:** immunotherapy, LPAS, LUAD, prognosis, TME

## Abstract

We employed single‐cell analysis techniques, specifically the inferCNV method, to dissect the complex progression of lung adenocarcinoma (LUAD) from adenocarcinoma in situ (AIS) through minimally invasive adenocarcinoma (MIA) to invasive adenocarcinoma (IAC). This approach enabled the identification of Cluster 6, which was significantly associated with LUAD progression. Our comprehensive analysis included intercellular interaction, transcription factor regulatory networks, trajectory analysis, and gene set variation analysis (GSVA), leading to the development of the lung progression associated signature (LPAS). Interestingly, we discovered that the LPAS not only accurately predicts the prognosis of LUAD patients but also forecasts genomic alterations, distinguishes between ‘cold’ and ‘hot’ tumours, and identifies potential candidates suitable for immunotherapy. PSMB1, identified within Cluster 6, was experimentally shown to significantly enhance cancer cell invasion and migration, highlighting the clinical relevance of LPAS in predicting LUAD progression and providing a potential target for therapeutic intervention. Our findings suggest that LPAS offers a novel biomarker for LUAD patient stratification, with significant implications for improving prognostic accuracy and guiding treatment decisions.

## 1 INTRODUCTION

For decades, lung cancer (LC) has persistently ranked as the foremost cause of cancer‐related mortality on a global scale, with both its incidence and mortality rates exhibiting a continual ascent.^1^ Among LC cases, non‐small‐cell lung cancer (NSCLC) represents a substantial majority, with lung adenocarcinoma (LUAD) emerging as its predominant histological subtype.^2^ Despite notable strides in LUAD treatment strategies, the 5‐year overall survival (OS) rate remains below 20% due to the belated diagnosis of a significant proportion of LUAD patients.^3^ Immunotherapy has heralded a paradigm shift in the treatment landscape of LUAD, affording therapeutic prospects to patients at advanced stages or those eligible for early surgical resection. However, the intrinsic immunological diversity within LUAD leads to a subdued response rate, with only a minority of patients deriving therapeutic benefits.^4^ The primary challenge in the realm of tumour immunotherapy lies in the identification of prognostic biomarkers that can gauge treatment efficacy and prognosticate patient outcomes. While certain biomarkers, such as programmed death‐ligand 1 (PD‐L1) expression and tumour mutation burden (TMB), have gained widespread clinical traction for predicting immunotherapeutic responses,^5^ they fall short in encapsulating the complete spectrum of the tumour microenvironment's (TME) heterogeneity. Hence, the exigency of crafting predictive models and unearthing novel biomarkers to prognosticate both clinical outcomes and therapeutic responses is palpable.

Within the gamut of LUAD subtypes, the most prevalent progression trajectory encompasses the sequential development of tumours, commencing with atypical adenomatous hyperplasia (AAH), advancing to adenocarcinoma in situ (AIS), then to minimally invasive adenocarcinoma (MIA), culminating in invasive adenocarcinoma (IAC) marked by conspicuous invasiveness. The clinical, pathological, and molecular attributes of these tumours lend credence to this stepwise evolutionary narrative.^6^ Conventional sequencing modalities, such as whole‐exome sequencing, have provided insights into the genomic landscape and immune microenvironmental characteristics during LUAD development, unmasking the pivotal roles of driver mutations in genes, such as EGFR, ERBB2, TP53 and BRAF.^7^, ^8^, ^9^ Nevertheless, comprehensive insights into the distribution of tumour cells and microenvironmental constituents throughout the process of LUAD progression remain a scarce commodity, necessitating the application of sequencing technologies for delineating cell‐type‐specific profiles.

Single‐cell RNA sequencing (scRNA‐seq) has emerged as an invaluable tool in demystifying the complexities of tumour heterogeneity and its evolutionary trajectory in LUAD, thereby facilitating the advent of precision oncology. scRNA‐seq has transformed our approach to dissecting the cellular landscape within resected NSCLC specimens, overcoming previous technical challenges that obscured our understanding of NSCLC heterogeneity.^10^ Recent strides in scRNA‐seq have led to the development of comprehensive single‐cell multi‐omics atlases for LUAD, unveiling the critical involvement of AT2 and basal cells in driving tumour progression.^11^, ^12^ In this study, we integrated and analysed scRNA‐seq data from two unique cohorts of LUAD patients. Our goal was to delineate the diversity within the TME across different invasion stages and identify key molecular changes throughout LUAD progression. We also developed a critical gene signature to predict disease trajectory and response to immunotherapy in LUAD. This model has been rigorously validated, demonstrating its potential to enhance personalized prognosis and guide clinical management in LUAD.

## 2 METHOD

### 2.1 Data set source

Utilizing The Cancer Genome Atlas (TCGA) database (https://portal.gdc.cancer.gov), we successfully obtained a substantial volume of data related to LUAD patients. This data include RNA sequencing, methylation data, copy number variation (CNV) data, mutation data and clinical characteristics. Additionally, we acquired two scRNA‐seq data sets (GSE189357 and GSE150938) from the Gene Expression Omnibus (GEO) database (http://www.ncbi.nlm.nih.gov/geo), comprising 4 AIS, 8 MIA, and 9 IAC samples. We also sourced five additional data sets from the GEO database, all containing clinical survival information, for model validation. These data sets are as follows: GSE13213^13^ (*n* = 119), GSE29016^14^ (*n* = 39), GSE30219^15^ (*n* = 86), GSE31210^16^ (*n* = 227) and GSE68465^17^ (*n* = 443). All transcript data used for building the model are stored in Table S1.

Furthermore, RNA sequencing data from patients receiving immunotherapy was obtained from the GEO website or the Tumor Immune Dysfunction and Exclusion (TIDE) database (http://tide.dfci.harvard.edu). The details of these data sets are as follows:

GSE35640: 65 melanoma patients participated in an immunological adjuvant trial involving the recombined MAGE‐A3 antigen.^18^

PRJEB25780: 57 patients with metastatic or recurrent gastric cancer receiving anti‐PD‐1 therapy.^19^

GSE94873: A large cohort of patients with advanced melanoma treated with tremelimumab.^20^

GSE78220: 28 cases of melanoma patients treated with anti‐PD‐1 therapy.^21^

GSE91061: 25 advanced melanoma patients treated with Nivolumab.^22^

PRJEB23709: 51 melanoma patients receiving PD‐1 and CTLA‐4 combination therapy.^23^

iMvigor210: 310 bladder cancer patients were treated with Atezolizumab.^24^

GSE135222: 27 advanced NSCLC patients were treated with anti‐PD‐1/PD‐L1 therapy.^25^

GSE100797: 27 stage IV melanoma patients were treated with adoptive T‐cell therapy.^26^

GSE126044: 16 NSCLC patients received PD‐1 therapy.^27^

GSE165252: 77 oesophageal adenocarcinoma patients were treated with neoadjuvant chemoradiotherapy combined with Atezolizumab.^28^

GSE173839: 105 breast cancer patients received Durvalumab in combination with olaparib and paclitaxel.^29^

Protein‐level data for LUAD was downloaded from the Clinical Proteomic Tumor Analysis Consortium (CPTAC) website (https://proteomics.cancer.gov/programs/cptac). The protein abundances were log2‐transformed and median‐centred for analysis.

These data resources were instrumental in our research, providing a comprehensive understanding of the molecular characteristics of LUAD patients and their responses to immunotherapy. To ensure data uniformity and comparability, we converted gene expression data into Transcripts Per Million (TPM) format and addressed potential batch effects using the ‘combat’ function within the ‘sva’ R package. Additionally, all data from the TCGA database, including large‐scale sequencing data from GEO, underwent log transformation to achieve a standardized data format before analysis initiation.

### 2.2 Cancer cell lines

Data pertaining to human cancer cell lines (CCLs) was procured from the Cancer Cell Line Encyclopedia (CCLE) project by the Broad Institute, accessible at https://portals.broadinstitute.org/ccle/. Additionally, genome‐wide CRISPR knockout screening data encompassing 739 cell lines, and 18,333 genes was collected from the Dependency Map (DepMap) portal, available at https://depmap.org/portal/. The dependency of genes in specific CCLs was assessed using CERES scores, with lower scores signifying a greater impact of these genes on cell growth and survival within the respective CCL. Furthermore, drug sensitivity data for CCLs was obtained from the Cancer Therapeutics Response Portal (CTRP) and the PRISM Repurposing dataset. CTRP contains sensitivity data for 481 compounds tested on 835 CCLs, while PRISM provides sensitivity data for 1448 compounds across 482 CCLs. Both data sets use AUC values to gauge drug sensitivity, with lower AUC values indicating higher responsiveness to treatment. Compounds with more than 20% missing data were excluded before imputation. Molecular data from the CCLE project were used for subsequent analyses of CTRP and PRISM data, as the CCLs in these data sets originated from the CCLE.

### 2.3 The detailed steps of the scRNA‐seq analysis

The original gene expression matrix underwent preprocessing with the Seurat R package (version 4.2.0).^30^ Genes had to demonstrate expression in a minimum of 10 cells within each sample to be included. Subsequently, subpar cells were filtered out based on specific criteria: those with more than 5000 or fewer than 200 expressed genes or cells with over 10% of unique molecular identifiers (UMIs) originating from the mitochondrial genome. The remaining high‐quality single‐cell transcriptome expression matrix was then integrated using the harmony R package. Subsequently, highly variable genes were selected for principal component analysis (PCA), and the top 30 significant principal components (PCs) were chosen for t‐distributed stochastic neighbour embedding (t‐SNE) dimension reduction for gene expression visualization. Differentially expressed genes (DEGs) within each cell subpopulation were identified using the “FindAllMarker” function, and cell types and subtypes were annotated based on the expression of established canonical marker genes for each cell type.

### 2.4 InferCNV and trajectory analysis

InferCNV was employed to infer tumour cell populations, using endothelial cells as the reference group. We set the parameter ‘k_obs_groups = 8’, which enabled us to categorize epithelial cells into eight distinct clusters. This stratification facilitated the identification and differentiation of copy number variations (CNVs) across each cluster, enhancing our understanding of the genomic landscape within the tumour. Subsequently, the Monocle2 algorithm was employed to perform developmental trajectory analysis using tumour cells inferred. A gene‐cell matrix extracted from UMI counts scaled within the Seurat subset was used as input. A new ‘cell data set’ function was used to create an object with an expression family parameter set to negative binomial size. Following dimension reduction and unit ordering, cell trajectories were inferred using default parameters.

### 2.5 Cell–cell interaction

CellChat^31^ was utilized to merge gene expression data and evaluate variations in proposed cell–cell communication modules. The default CellChatDB was used as the ligand–receptor database in accordance with the standard CellChat process. Cell type‐specific interactions were deduced by detecting overexpressed ligands or receptors within a specific cell group, followed by the identification of intensified ligand–receptor interactions when these ligands or receptors were overexpressed.

### 2.6 SCENIC analysis

The activity of gene regulatory networks is inferred using the R software package Scenic. The activity of each regulator in single cells is scored using default settings and the following cisTarget databases: hg38_refseq‐r80_500bp_up_and_100bp_down_tss.mc9nr.feather and hg38_refseq‐r80_10kb_up_and_down_tss.mc9nr.feather.

### 2.7 Determination of key genes in Cluster 6 subgroup

Weighted gene co‐expression network analysis (WGCNA) was utilized to construct a co‐expression network for TCGA‐LUAD data set. An optimal soft threshold power β was meticulously determined to conform to the criteria of scale‐free network topology. Subsequently, the weighted adjacency matrix was transformed into a topological overlap matrix (TOM), followed by the computation of the corresponding dissimilarity measure (1‐TOM). Module identification was performed employing the dynamic tree‐cutting method. In order to pinpoint modules significantly associated with ssGSEA‐Cluster6, the module exhibiting the paramount correlation was selected for in‐depth analysis.

### 2.8 Building the lung progression associated signature (LPAS)

Univariate Cox regression analysis was employed to assess the impact of key genes within the cluster 6 subgroup on the survival status of LUAD. A significance threshold of 0.05 was set to minimize the possibility of overlooking important factors. Subsequently, the LASSO Cox regression method was used to reduce the pool of candidate genes, ultimately creating the most effective survival signature. Predictive performance was evaluated using receiver operating characteristic (ROC) curves, and an area under the curve (AUC) value exceeding 0.65 was considered indicative of outstanding performance.

### 2.9 Mutation landscape

Genomic alterations, such as recurrently amplified and deleted regions, were identified using GISTIC 2.0 analysis. The R package ‘maftools’^32^ was employed to calculate the tumour mutational burden (TMB).

### 2.10 Differences in the TME and drug inference

Seven distinct immune infiltration algorithms were used to comprehensively evaluate the composition of immune cells in various LPAS groups. Heatmaps were then applied to visually illustrate the nuanced differences in immune cell infiltration across these LPAS groups. Furthermore, the specialized functionalities of the ‘estimate’ R package^33^ were carefully leveraged to quantify immune scores, stromal scores, and ESTIMATE scores for TCGA‐LUAD patients, facilitating a comprehensive assessment of the TME and its potential implications. For the identification of potentially effective chemotherapeutic agents within different LPAS groups, the predictive capabilities of the ‘oncoPredict’ and ‘pRRophetic’ R packages^34^, ^35^ were extensively utilized. This tool enabled accurate predictions of suitable therapeutic interventions, contributing to a more informed treatment strategy.

### 2.11 Enrichment analysis

A gene set enrichment analysis was carried out using 50 hallmark pathways from the Molecular Signatures Database (MSigDB). To estimate pathway activity for each cell type, Gene Set Variation Analysis (GSVA) was conducted for individual cells. Subsequently, the average gene expression levels for each cell subtype were calculated using the default GSVA package settings. Differences in activity scores were then used to measure variations in pathway activity among different cell subtypes.

### 2.12 Clinical specimen collection and RNA sequencing

The collection of tissue samples has received ethical approval from the Medical Ethics Committee of the First Affiliated Hospital of Nanjing Medical University. These samples, categorized as AIS, MIA, or IAC by pathology experts, are obtained on the day of the surgery and are then sent to Oncocare Inc. (Suzhou, China) for RNA sequencing.

### 2.13 SubMap validation

The assessment of shared characteristics between two groups is conducted using the unsupervised SubMap method. Significance is determined by an adjusted *p*‐value below 0.05, indicating a substantial degree of similarity. Subtype consistency between the validation and discovery cohorts was evaluated through the SubMap approach, and the outcomes were then visualized using the ComplexHeatmap package.

### 2.14 Cell lines culture

A549 and H1299 cells (human lung adenocarcinoma cell lines) were sourced from the Cell Resource Center of Shanghai Life Sciences Institute. They were cultured in a medium consisting of F12K or RPMI‐1640 (Gibco BRL, USA) supplemented with 10% fetal bovine serum (FBS), 1% streptomycin and penicillin (Gibco, Invitrogen, Waltham, MA, USA). The cells were maintained at 37°C under conditions of 5% CO2 and 95% humidity.

### 2.15 Cell transfection

The knockdown of PSMB1 was achieved through the use of small‐interfering RNA (siRNA). Cells were seeded in a six‐well plate at 50% confluence and were transfected with both a negative control (NC) and the knockdown siRNA (siPSMB1). All transfection procedures were performed using Lipofectamine 3000 (Invitrogen, USA).

### 2.16 Cell‐counting kit‐8 experiment (CCK‐8)

In 96‐well plates, a cell suspension containing 3 × 10^3^ cells per well was dispensed. Subsequently, the plates were incubated for 2 h at 37°C under dark conditions with the addition of 10 mL of CCK‐8 labelling reagent (A311‐01, Vazyme) in each well. The absorbance of the cells at 450 nm was quantified using an enzyme‐linked reader (A33978, Thermo) at 0, 24, 48, 72 and 96 h in order to determine cell viability.

### 2.17 Colony formation

We transfected 1 × 10^3^ cells into each well of a six‐well plate and kept the cells alive for 14 days. Before Crystal violet (Solarbio, China) staining, the cells were washed twice with PBS and fixed for 15 min in 4% paraformaldehyde.

### 2.18 Wound‐healing assay

In six‐well plates, transfected cells were seeded and cultured in a cell incubator until they reached 95% confluence. In each well, a sterile 20 μL plastic pipette tip was used to gently create a single straight line across the monolayer of cells, resulting in the formation of a “wound.” Subsequently, the wells were gently washed twice with phosphate‐buffered saline (PBS) to remove any unattached cells and debris. Finally, photographs of the scratch wounds were taken using ImageJ software at two time points: 0 and 48 h after the scratch was made, and the width of the scratches was measured.

### 2.19 Transwell assay

In the transwell assay, studies on cell invasion and migration were conducted. The top chambers of 24‐well plates were filled with A549 and H1299 cells (2 × 10^5^) that had been treated, and they were incubated for 48 h. To assess the cells' invasive and migratory capabilities, the top section of the plate was either pre‐coated with Matrigel solution (BD Biosciences, USA) or left untreated. Following the removal of cells from the top surface, the remaining cells in the bottom layer were fixed with 4% paraformaldehyde and stained with 0.1% crystal violet (Solarbio, China).

### 2.20 Statistical analysis

Data processing, statistical analysis and visualization were performed using R 4.2.0 software. Subtype‐specific OS was estimated and compared using the Kaplan–Meier method and log‐rank test. Differences in continuous variables between the two groups were evaluated using the Wilcoxon test or *t*‐test. Categorical variables were analysed using the chi‐squared test or Fisher's exact test. The false discovery rate (FDR) method was applied to correct *p*‐values. Correlations between variables were examined using Pearson correlation analysis. All *p*‐values were calculated using a two‐tailed approach, with statistical significance defined as *p* < 0.05.

## 3 RESULTS

### 3.1 The scRNA profiling of LUAD

**FIGURE 1:**
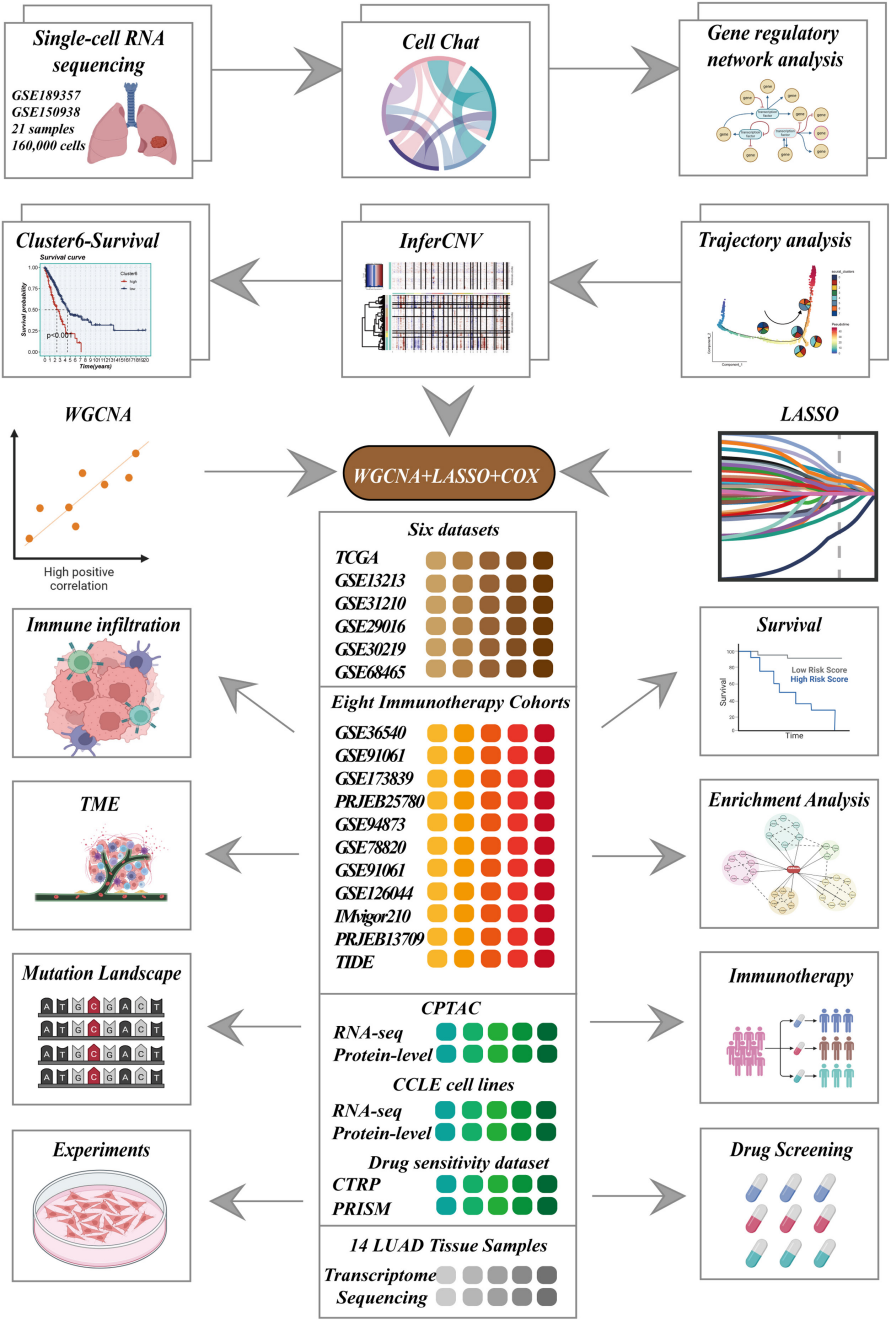
Flowchart of the analysis.

**FIGURE 2:**
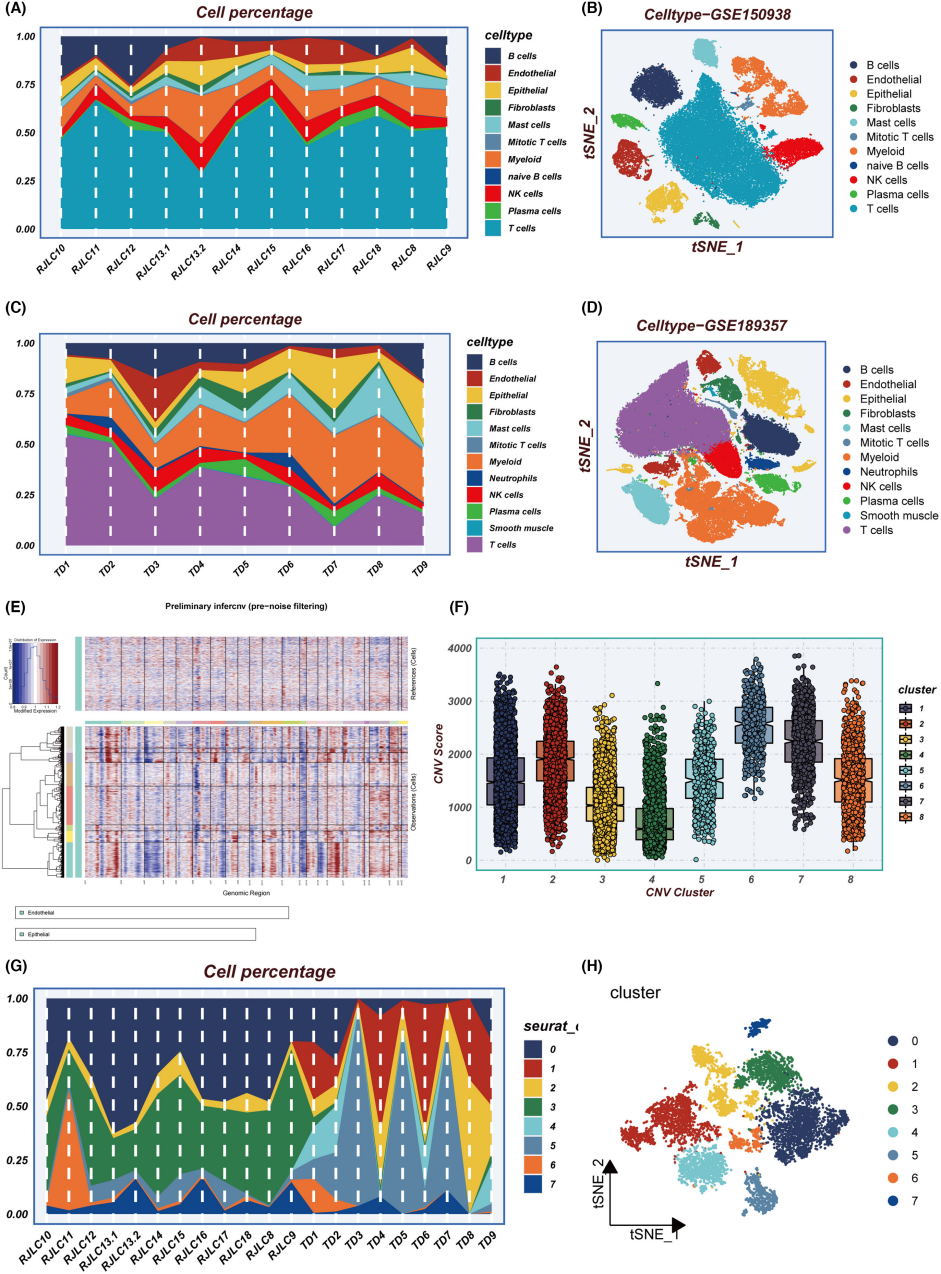
Integrating multiple single‐cell data sets for inference and re‐clustering of tumour cells. (A) Percentage of each cell type in different samples in GSE150938. (B) tSNE plot showing the distribution of cells in GSE150938. (C) Percentage of each cell type in different samples in GSE189357. (D) tSNE plot showing the distribution of cells in GSE189357. (E) Heatmap showing the situation of the InferCNV analysis. (F) Distribution of CNVs for eight clusters. (G) Percentage of tumour subgroups in different samples. (H) tSNE plot showing the distribution of tumour subgroups. CNV, copy number variation; tSNE, t‐distributed stochastic neighbour embedding.

In the experimental framework illustrated in Figure 1, we employed specific identification markers to classify all sampled cells from the data sets GSE150938 and GSE189357 into 12 distinct cell types, as demonstrated in the t‐SNE plots (Figure 2A–D). Notably, the proportion of cell types varied among different patients. Focusing on Epithelial and Endothelial cells, we conducted an exhaustive analysis of CNVs in each chromosome across all cells using the InferCNV algorithm, as depicted in Figure 2E. Our analysis indicated that the majority of epithelial cells exhibited higher CNV levels than endothelial cells. Figure 2F presents the variance in CNV scores among eight identified cell clusters, highlighting that Clusters 4 and 5 manifested relatively lower CNVs. These clusters were designated normal cells, whereas the remaining clusters were classified as tumour cells. Subsequently, all tumour cells were re‐clustered. This process resulted in the identification of eight distinct clusters, as illustrated in Figure 2G,H.

### 3.2 Biological characteristic analysis of tumour clusters

**FIGURE 3:**
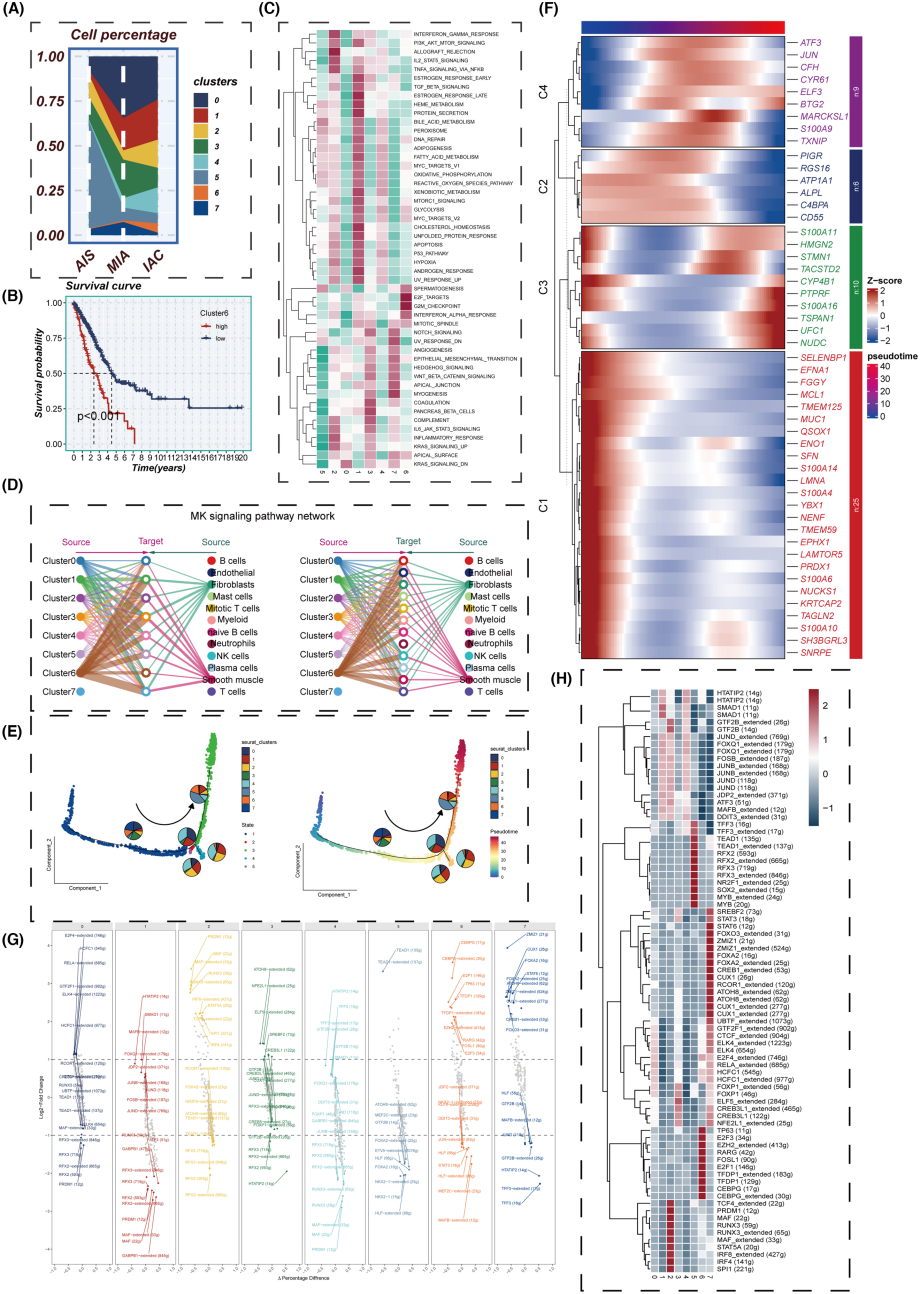
Cell subgroup analysis. (A) Changes in cell subgroup percentages from AIS to IAC, (B) KM survival curve in Cluster 6, (C) GSVA enrichment analysis, (D) MK signalling network, (E) cell subgroup trajectory analysis, (F) heatmap showing the expression of differential genes over pseudotime, (G) transcription factor regulatory networks specifically upregulated and downregulated in each tumour cluster, (H) heatmap presenting the distribution of gene regulatory networks in different clusters. GSVA, gene set variation analysis; IAC, invasive adenocarcinoma.

Figure 3A shows the changes in the proportion of the eight clusters as LUAD progresses from AIS to IAC, revealing that Cluster 6 generally shows an increasing trend. KM survival analysis of the eight clusters reveals that only the signature composed of specific genes within Cluster 6 has predictive significance for LUAD prognosis (Figure 3B). GSVA enrichment analysis found that genes in Cluster 6 are mainly enriched in pathways such as E2F targets and G2M checkpoints, which are largely associated with the cell cycle (Figure 3C). The MK signalling pathway network illustrated the internal relationships between Clusters, Target and Source, and the results showed that Cluster 6 has the strongest connections with various targets (Figure 3D). The MK signalling pathway typically represents a pivotal role in cell migration and tumour progression.^36^ Trajectory analysis indicated that the proportion of Cluster 6 gradually increases (Figure 3E). In pseudotime, we identified the top 50 highly variable genes (Figure 3F). Based on their expression in pseudotime, we grouped them into four gene clusters. Subsequently, the Scenic package was employed to evaluate differences in the expression levels of transcription factors (TFs) in epithelial cells. Interestingly, it was observed that transcription factors such as E2F1, E2F3, TP63 and EZH2 were significantly upregulated in cluster 6 (Figure 3G,H). Multiple studies have indicated that the abnormal expression or activity of these transcription factors may contribute to the promotion of tumour development and progression in LUAD.^37^, ^38^, ^39^ In summary, we believe that key genes affecting the progression of LUAD exist within cluster 6.

### 3.3 Building a highly robust LPAS

**FIGURE 4:**
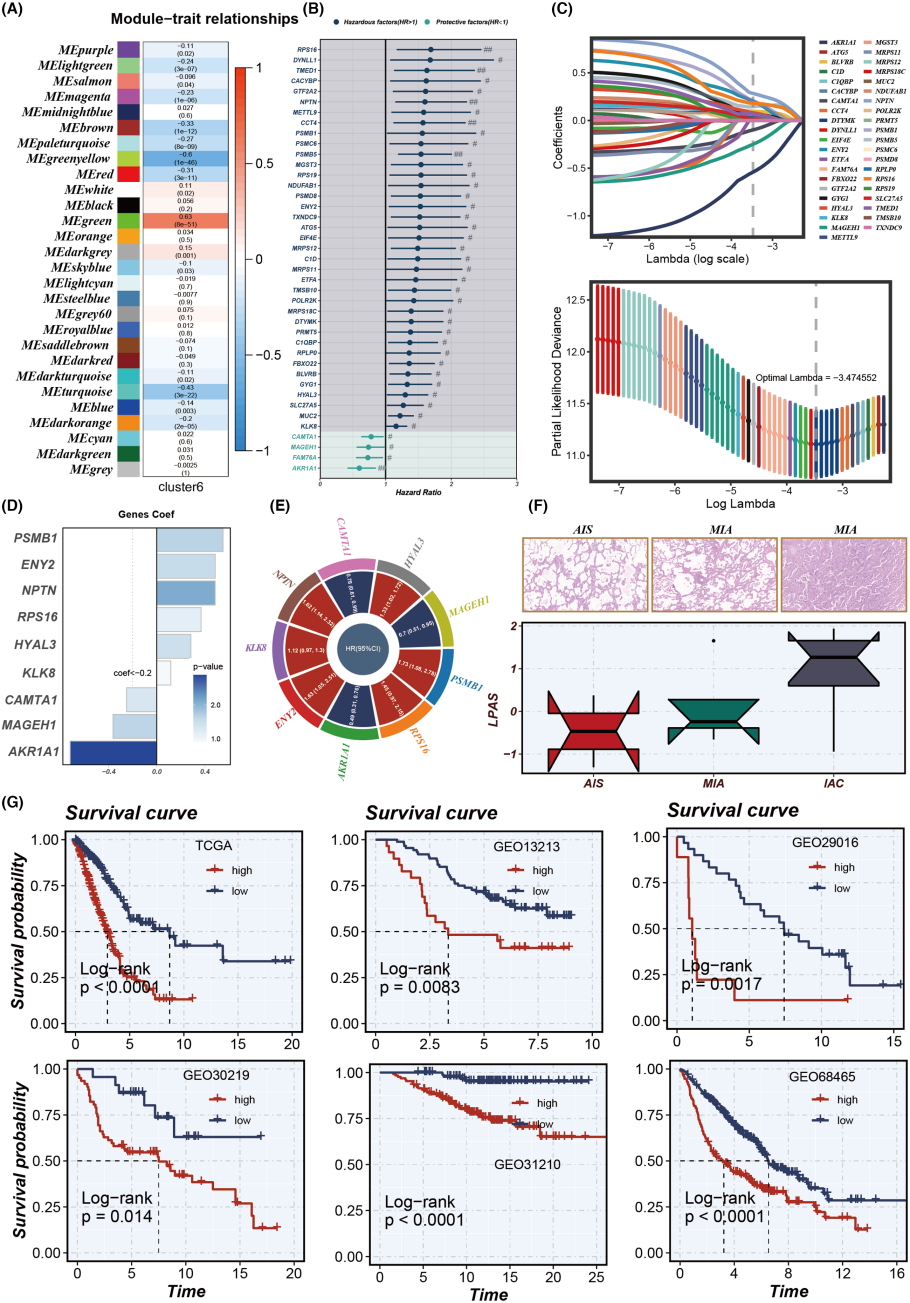
Model construction. (A) WGCNA was utilized to identify gene modules associated with Cluster 6. (B) The results of univariate Cox analysis of module genes. (C) LASSO regression was employed to select model genes. (D, E) Nine model genes were identified through multivariate Cox regression analysis, with each gene assigned distinct coefficients and hazard ratios (HRs). (F) Comparison of LPAS score differences in AIS, MIA and IAC. (G) KM survival curves in TCGA, GSE13213, GSE29016, GSE30219, GSE31210 and GSE68465. AIS, adenocarcinoma in situ; IAC, invasive adenocarcinoma; LPAS, lung progression associated signature; MIA, minimally invasive adenocarcinoma; WGCNA, weighted gene co‐expression network analysis.

Through WCGNA co‐expression analysis, all genes were divided into 28 modules (Figure 4A). Analysis revealed that the green gene module has the highest correlation (0.63) with Cluster 6. A univariate analysis of genes in the green module yields 41 genes that have some predictive significance for the prognosis of TCGA‐LUAD patients (Figure 4B). Using LASSO and COX regression, these 41 genes were analysed again, resulting in nine independent risk factors (PSMB1, ENY2, NPTN, RPS16, HYAL3, KLK8, CAMTA1, MAGEH1 and AKR1A1; Figure 4C). Based on these nine genes, a prognostic model (LPAS) for LUAD was constructed (Figure 4D,E). After performing transcript sequencing for 14 LUAD samples and calculating their LPAS scores, it was found that as the tumour progresses from AIS to MIA, and then to IAC, the LPAS scores gradually increase. This suggests that the more mature the tumour development is, the more malignant cells there are, and the higher the LPAS score becomes (Figure 4F).

### 3.4 Model evaluation and validation

**FIGURE 5:**
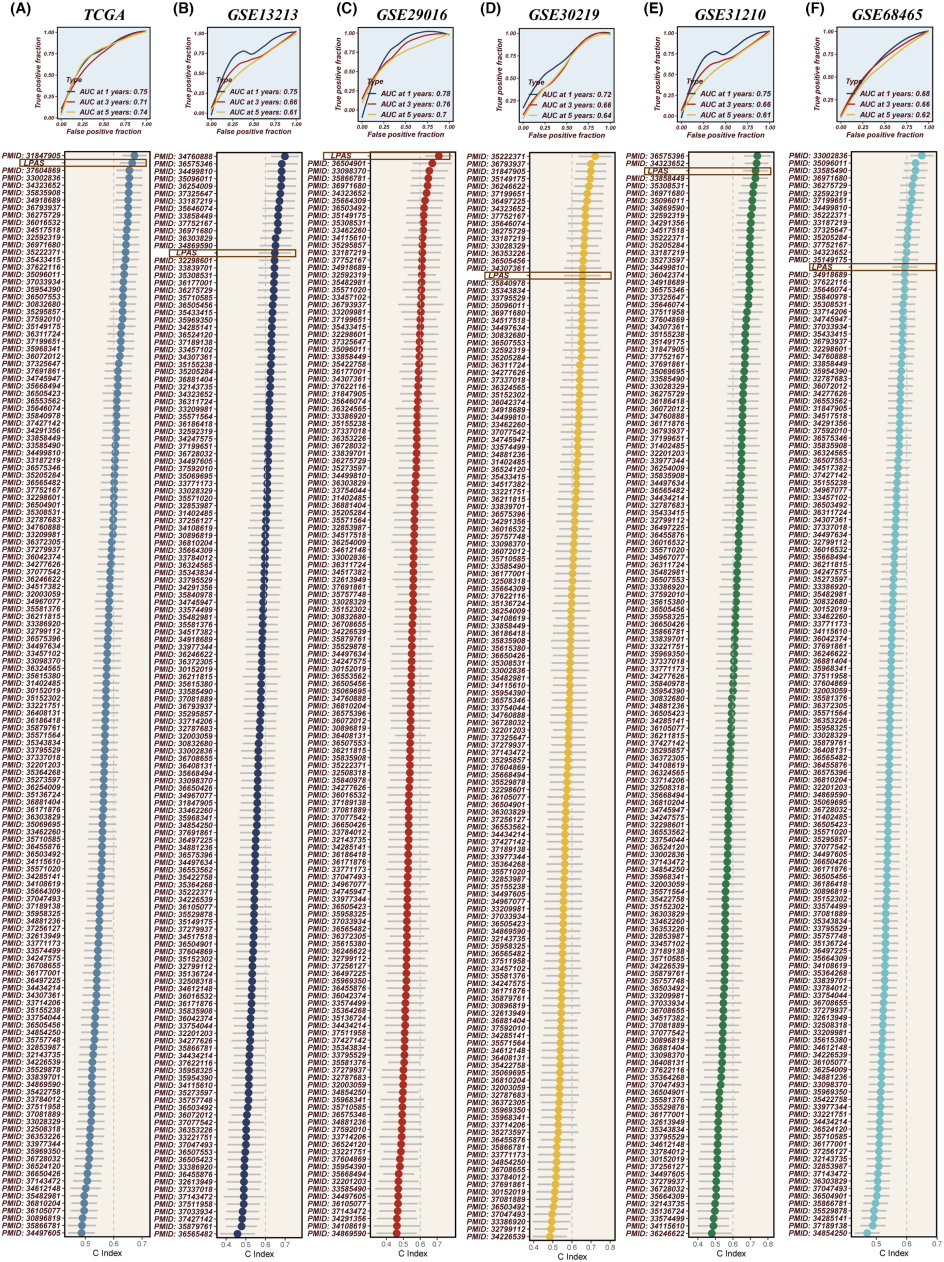
Evaluation of the LPAS. (A–F) The ROC curves of LPAS in the TCGA, GSE13213, GSE29016, GSE30219, GSE31210 and GSE68465 data sets; compared to 144 previously published models on LUAD, LPAS demonstrates superior prognostic efficacy. LPAS, lung progression associated signature; LUAD, lung adenocarcinoma; ROC, receiver operating characteristic.

Patients with high LPAS scores had noticeably worse prognoses for LUAD, a result that is consistent across six data sets: TCGA, GSE13213, GSE29016, GSE30219, GSE31210 and GSE68465 (Figure 4G). The AUC values for 1‐year, 2‐year, 3‐year, 4‐year and 5‐year OS demonstrated the good predictive performance of the LPAS signature in the TCGA (0.713, 0.719, 0.695, 0.693, 0.675), GSE13213 (0.752, 0.622, 0.661, 0.628, 0.608), GSE29016 (0.783, 0.779, 0.763, 0.797, 0.703), GSE30219 (0.720, 0.642, 0.656, 0.641, 0.652), GSE31210 (NA, 0.906, 0.847, 0.842, 0.793) and GSE68465 (0.677, 0.677, 0.669, 0.660, 0.629) data sets(Figure 5A–F). In order to further test the prognostic performance of the LPAS signature score, we included 144 signatures and compared the C‐index in the TCGA, GSE13213, GSE29016, GSE30219, GSE31210 and GSE68465 data sets. The results found that in the TCGA, GSE13213, GSE29016, GSE30219, GSE31210 and GSE68465 data sets, our LPAS feature performed better than most other previously published signatures(Figure 5A–F).

### 3.5 LPAS is closely related to immunity

**FIGURE 6:**
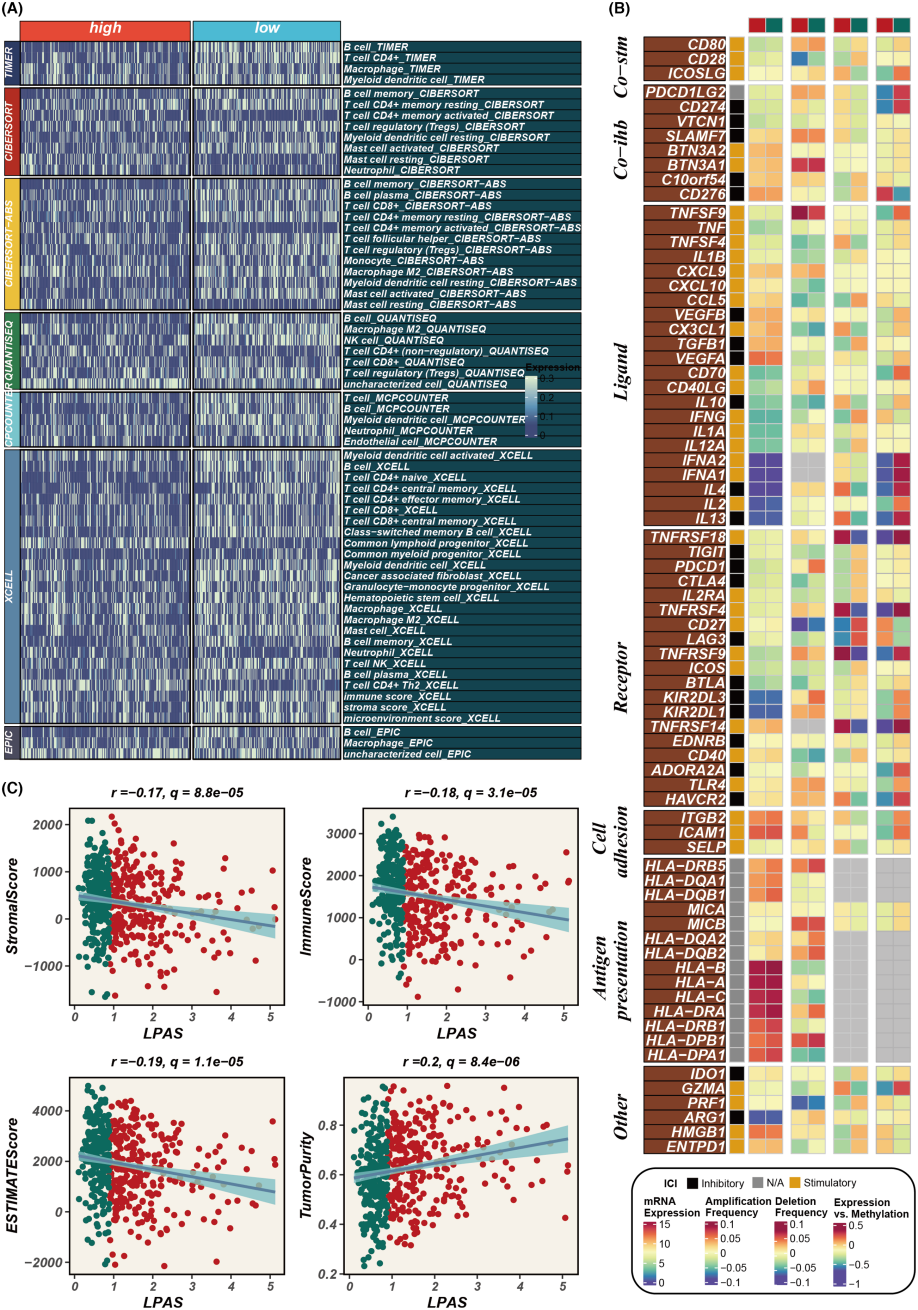
Immune infiltration status in high and low LPAS. (A) Heatmap reflects the difference in immune infiltration scores between high and low LPAS groups. (B) Correlation analysis of LPAS and immune modulators. (C) Scatter plots elucidate the correlation between LPAS score and stromal score, immune score, ESTIMATE score and tumour purity. LPAS, lung progression associated signature.

**FIGURE 7:**
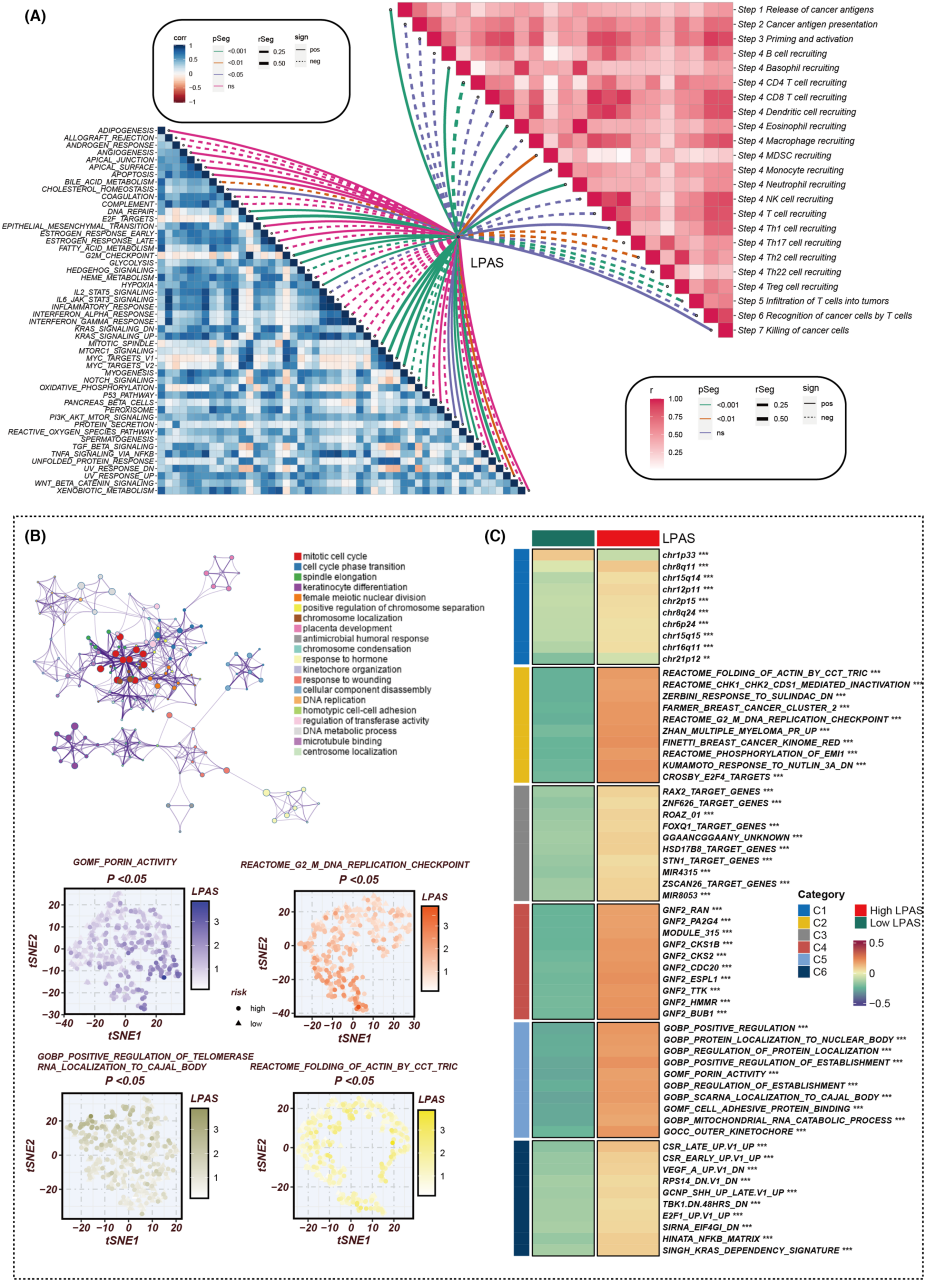
Biological characteristics of LPAS in the TCGA data set. (A) Heatmap showing the correlation between LPAS and hallmark signalling pathways as well as the cancer immune cycle. (B‐C) Differences in pathway enrichment between high and low LPAS groups. LPAS, lung progression associated signature.

In order to explore the immune status reflected by the LPAS score, we analyzed the association between the LPAS score and immune infiltrating cells and immune checkpoints. Using seven methods, the immune infiltration scores in the TCGA dataset were calculated. A heatmap shows that the degree of immune cell infiltration is higher in the group with low‐LPAS scores (Figure 6A). Further analysis revealed that the LPAS score is negatively correlated with matrix scores, immune scores, and ESTIMATE scores, while it is positively correlated with tumor purity (Figure 6C). Analysis of the correlation of the LPAS score with immune modulators found that the level of immune regulatory factors was lower in the group with high‐LPAS scores, suggesting that this group has a higher level of immune suppression (Figure 6B). We further studied the correlation between the LPAS and signaling pathways as well as the cancer‐immune cycle. Results showed that the LPAS is positively correlated with many signaling pathways and the cancer‐immune cycle. In the TCGA dataset, the level of immune infiltrating cells and immune modulators was high, suggesting that this group has an inflammatory but relatively immune‐promoting microenvironment, and this was precisely the potential beneficiary of immunotherapy (Figure 7A).

### 3.6 Enrichment analysis

To elucidate the immune‐related characteristics distinguishing the high‐ and low‐LPAS groups, we explored their underlying biological mechanisms in depth. Notably, the LPAS signature score exhibited a strong positive correlation with several key processes: porin activity, the G2/M DNA replication checkpoint, the folding of actin mediated by the CCT/TRIC complex, and the positive regulation of telomerase RNA localization to the Cajal body. These findings underscore the pronounced disparities between the two LPAS signature score groups in terms of oncogenic pathways and immune pathways (Figure 7B,C). Concurrently, the DEGs between the high‐ and low‐LPAS groups were predominantly enriched in the cell cycle pathway.

### 3.7 Immune checkpoints and potential drug targets

**FIGURE 8:**
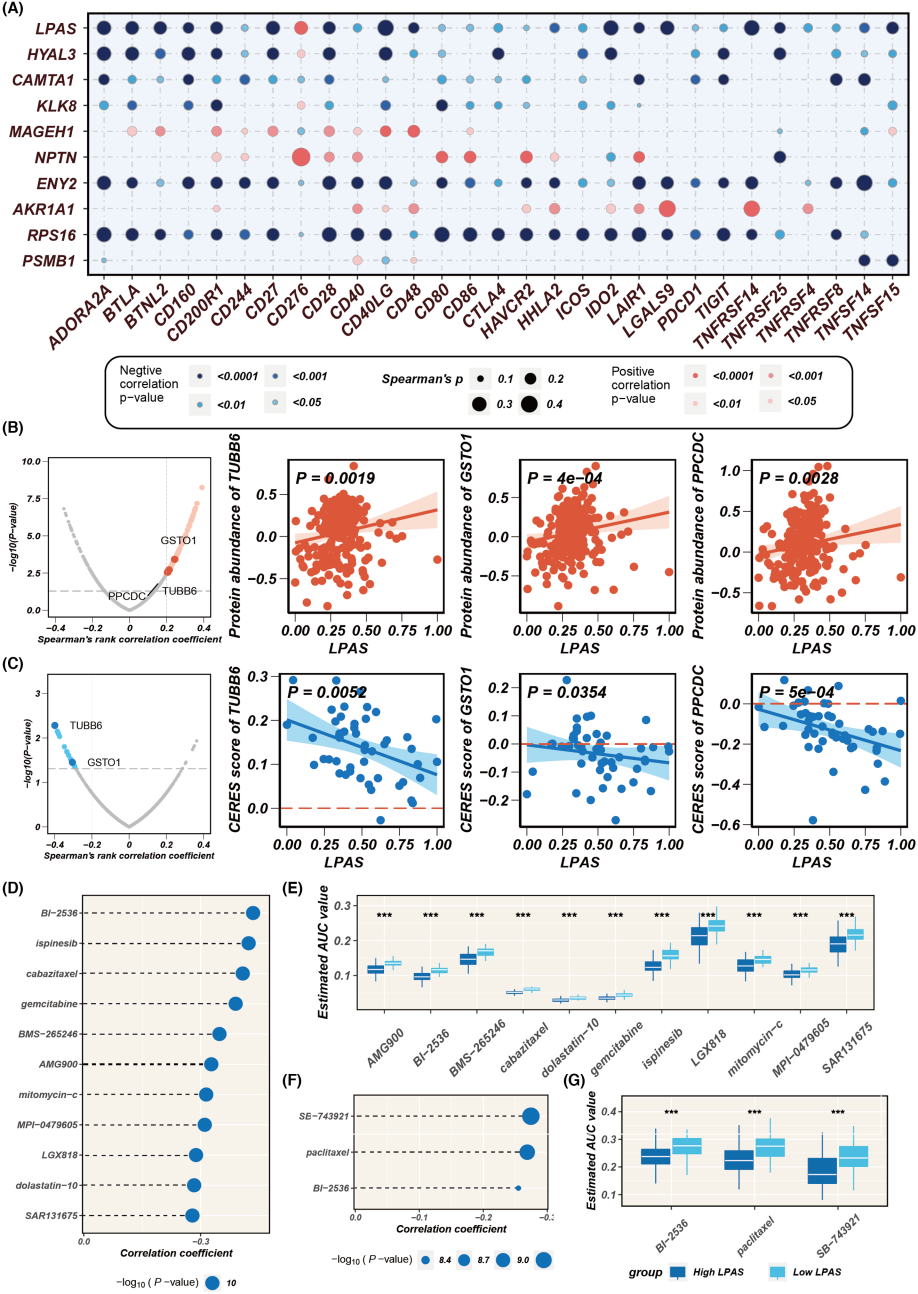
Immune checkpoint and drug target analysis. (A) Bubble plot showing the correlation between LPAS score and its model genes with common immune checkpoints. (B) Volcano plot and scatter plots demonstrating the correlation between LPAS score and the expression of drug target proteins. (C) Volcano plot and scatter plot of Spearman's correlations and significance between LPAS and CERES score of drug targets. (D) Spearman correlations for 11 CTRP‐derived compounds. (E) Results of differential drug response analysis for 11 CTRP‐derived compounds. (F) Spearman correlations for three PRISM‐derived compounds. (G) Results of differential drug response analysis for three PRISM‐derived compounds. CTRP, Cancer Therapeutics Response Portal; LPAS, lung progression associated signature.

In analysing the correlation between the LPAS score and model genes and common immune checkpoints, it was found that the LPAS score was negatively correlated with the majority of immune checkpoints, with the exception of CD276. MAGEH1, NPTN and AKR1A1 showed positive correlation with many immune checkpoints (Figure 8A). In search of potential target candidates, we analysed target information for numerous compounds, identifying three genes: TUBB6, GST01 and PPCDC. The protein abundance of TUBB6, GST01 and PPCDC positively correlated with LPAS, whereas their CERES scores negatively correlated with LPAS, suggesting that inhibiting the function of these three genes in patients with high‐LPAS scores may yield positive treatment results (Figure 8B,C). Based on drug response data from CTRP and PRISM, we sought potential therapeutic drugs for the high‐LPAS score group. We firstly performed a drug response difference analysis to identify compounds with lower estimated AUC values in the high‐LPAS score group. Then, using Spearman correlation analysis between the AUC values and the LPAS scores, we filtered out compounds with negative correlation coefficients. Following this analysis, we identified 11 compounds derived from CTRP (BI‐2536, ispinesib, cabazitaxel, gemcitabine, BMS‐265246, AMG900, mitomycin‐c, MPI‐0479605, LGX818, dolastatin‐10 and SAR131675) and three compounds from PRISM (SE‐743921, paclitaxel and BI‐2536). All these compounds have lower estimated AUC values in the high‐LPAS score group and are negatively correlated with LPAS (Figure 8D–G).

### 3.8 LPAS predicts genomic alterations

**FIGURE 9:**
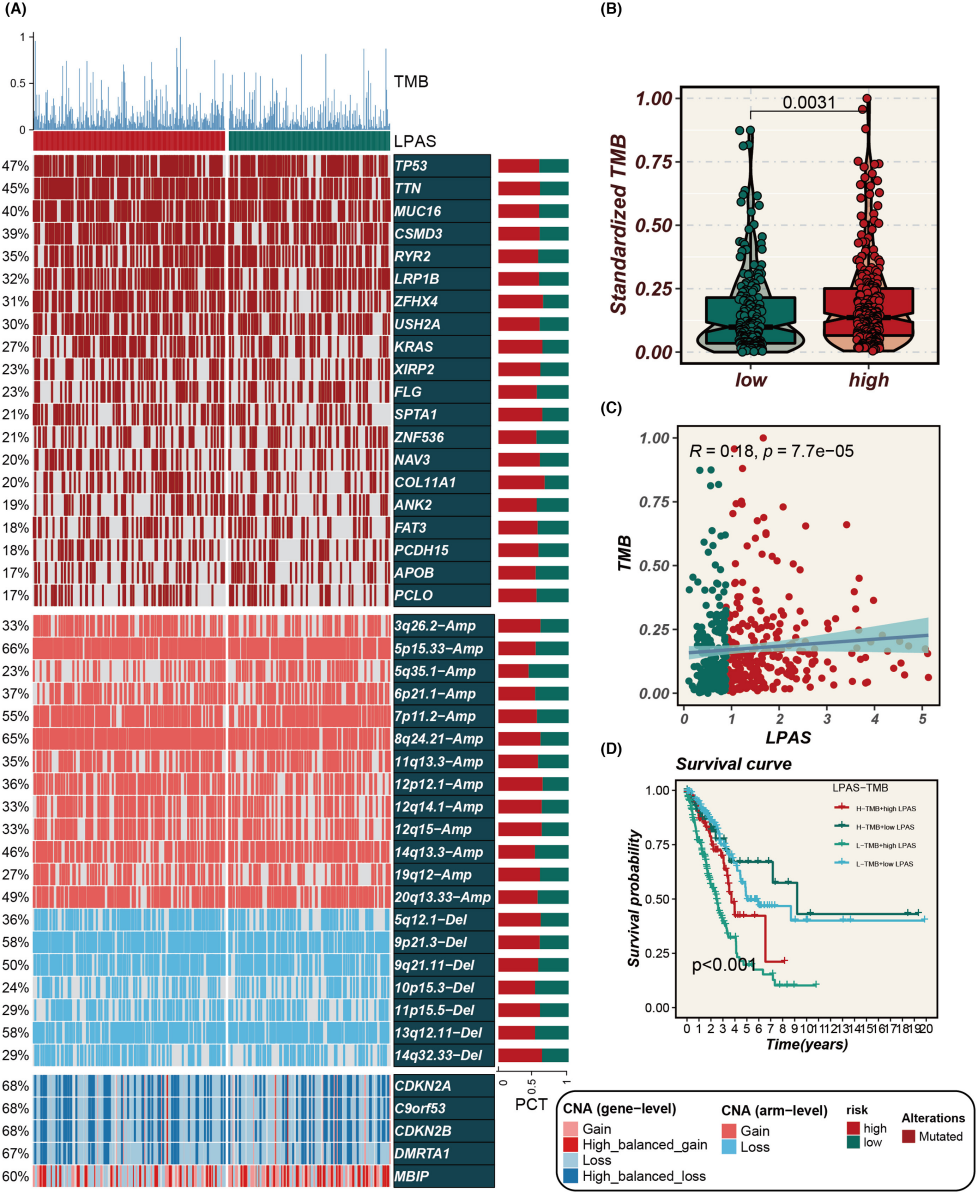
LPAS can predict genomic alterations. (A) Heatmap showing gene mutation status in high and low LPAS groups. (B) Differences in standardized TMB between high and low LPAS groups. (C) Correlation analysis between LPAS score and TMB. (D) Survival curves show the difference in survival among four subgroups (high LPAS and high TMB, high LPAS and low TMB, low LPAS and high TMB, low LPAS and low TMB). LPAS, lung progression associated signature; TMB, tumour mutation burden.

Different frequently altered chromosomes existed in the two LPAS signature score groups, with specific altered regions shown in Figure 9A. The TMB was higher in the group with high‐LPAS scores, and the LPAS score was positively correlated with the TMB score (Figure 9B,C). Survival analysis indicated that the prognosis is worse for the group with low TMB and high LPAS scores (Figure 9D).

### 3.9 Immune therapy cohort validation

**FIGURE 10:**
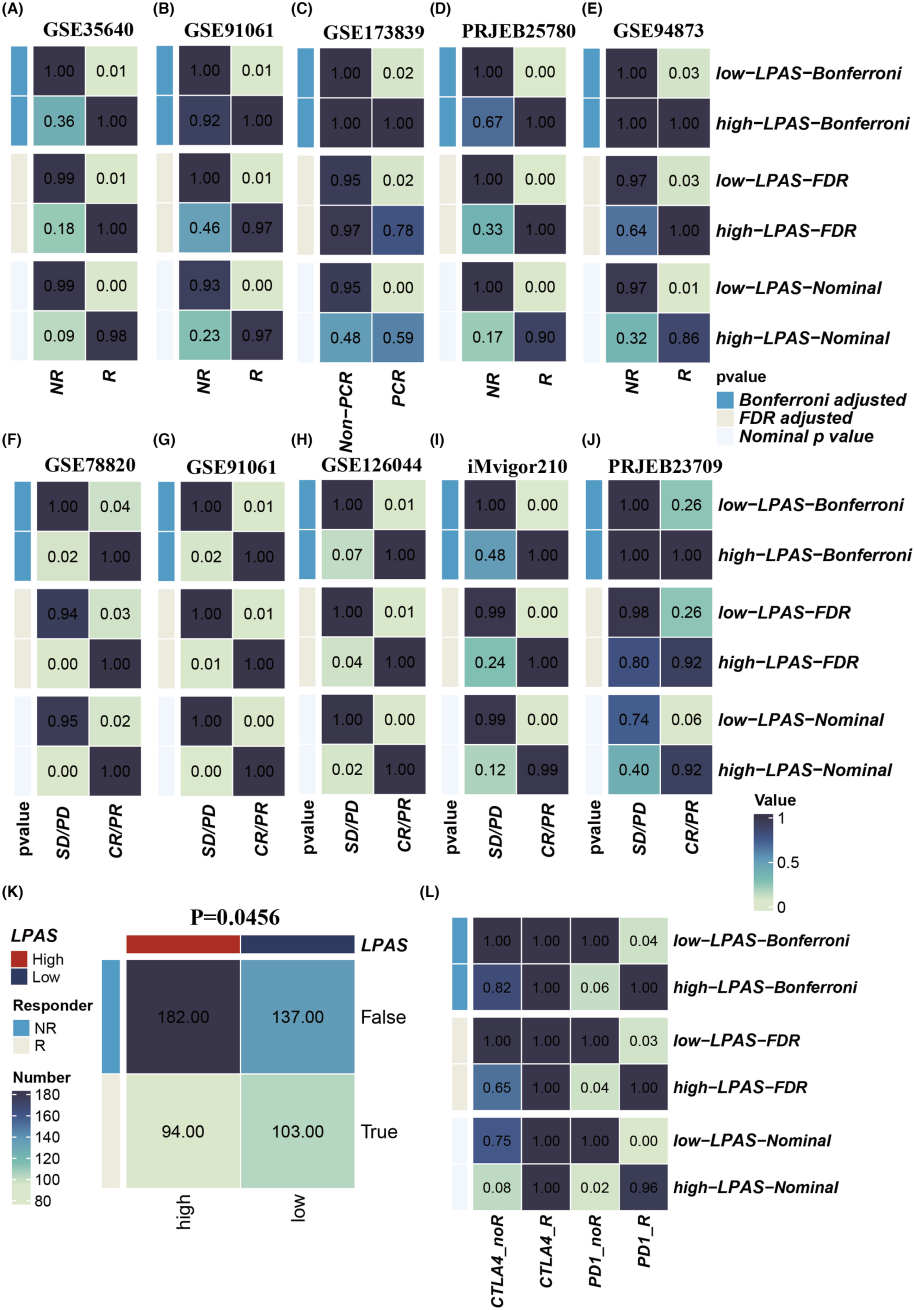
LPAS can predict immunotherapy response. (A–J) Immunotherapy response in high and low LPAS groups in 10 immune therapy cohorts. (K) TIDE algorithm predicting the relationship between LPAS and immune therapy response. (L) Submap predicting the relationship between LPAS and immune therapy response. LPAS, lung progression associated signature; TMB, tumour mutation burden.

For the purpose of identifying the predictive ability of the LPAS signature score for the efficacy of immunotherapy, we conducted validations in multiple immunotherapy data sets. The SubMap analysis results suggested that in all 10 immunotherapy cohorts, patients with low LPAS scores showed better immune responses (Figure 10A–J). The TIDE algorithm indicated that in the TCGA cohort, a low LPAS score is associated with a better immune response (Figure 10K). SubMap analysis suggested that in the TCGA cohort, a low LPAS score is associated with better anti‐CTLA‐4 and anti‐PD‐1 immune responses (Figure 10L).

### 3.10 PSMB1 may serve as a therapeutic target for LUAD

**FIGURE 11:**
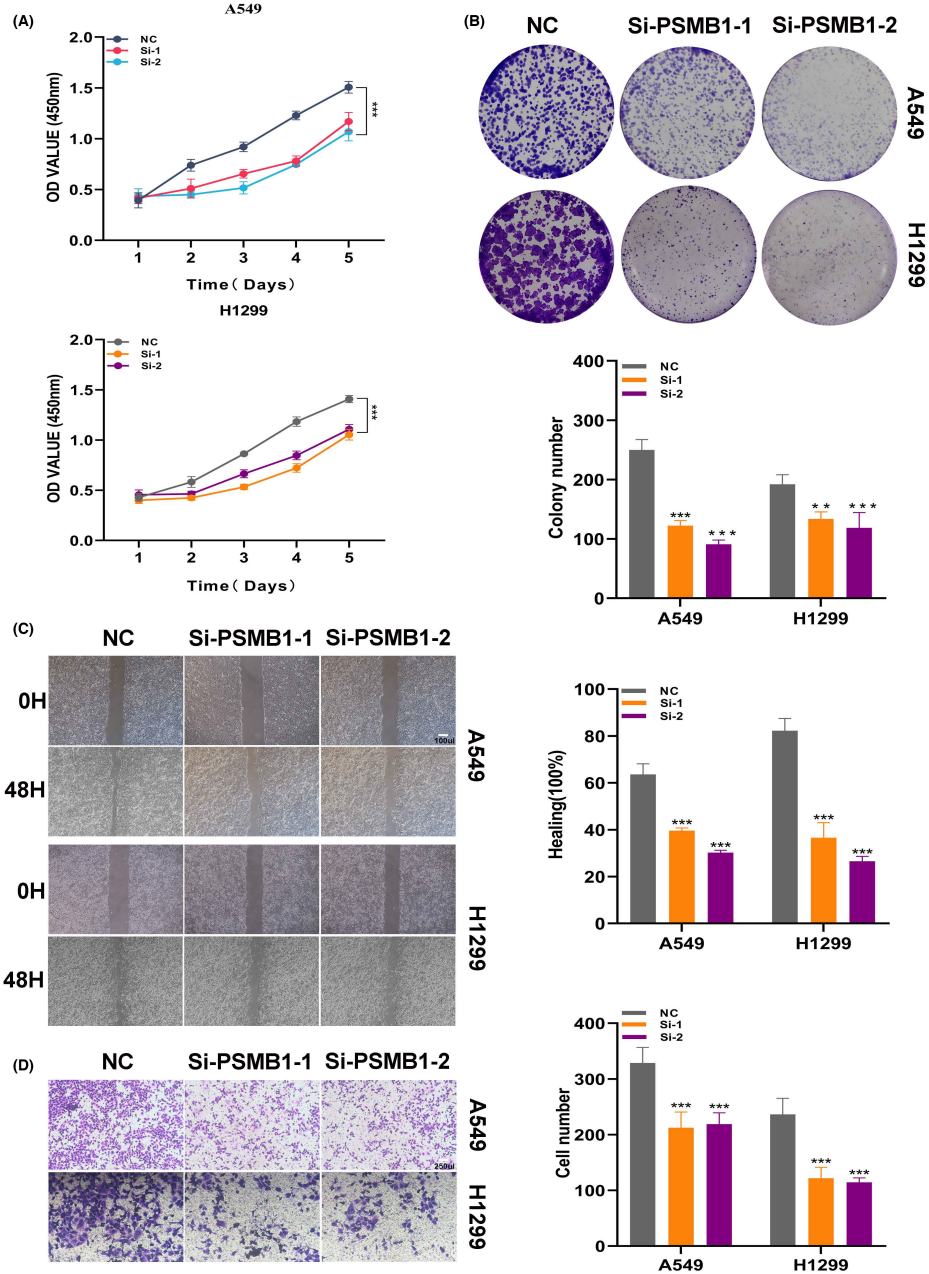
Experimental validation demonstrates that PSMB1 has a tumour‐promoting effect. (A, B) CCK‐8 detection and colony formation assays show that knockdown of PSMB1 expression significantly suppressed the proliferation and DNA replication of LUAD cells. (C) Wound‐healing assay to evaluate the impact of si‐PSMB1 transfection on the migratory ability of A549 and H1299 cells. (D) Transwell assay to examine the migration capacities of transfected A549 and H1299 cells. LUAD, lung adenocarcinoma.

Among all model genes, PSMB1 has the highest HR value, indicating its stronger impact on LUAD prognosis. To validate the carcinogenic role of PSMB1 in LUAD, we used siRNA to inhibit the expression of PSMB1 in A549 and H1299 cells. The CCK‐8 and colony formation experiments indicated that inhibiting PSMB1 can significantly suppress the proliferation and DNA replication capability of LUAD cells (Figure 11A,B). A wound‐healing assay was used to measure cell migration capability. The results showed that, compared with the control group, the wound‐healing rate of A549 and H1299 cells with PSMB1 knockdown was significantly reduced (Figure 11C). Transwell experiments showed that the number of cells invading the lower chamber decreased after PSMB1 knockdown (Figure 11D). These results suggested that PSMB1 plays a tumour‐promoting role in LUAD cells, and its hazardous role in LPAS has been validated.

## 4 DISCUSSION

LUAD, a primary pathological subtype of lung cancer (LC), can be stratified into distinct stages: AIS, MIA and IAC, reflecting a sequence of tumour progression.^40^, ^41^, ^42^ Each stage significantly impacts patient prognosis, yet the mechanisms driving the progression from AIS to MIA to IAC remain elusive. Recent advancements in single‐cell‐sequencing technology have greatly enhanced our understanding of tumour heterogeneity at both cellular and molecular levels, improving insights into tumour initiation, progression, metastasis and prognosis.^43^ This technological evolution has also been instrumental in identifying potential therapeutic targets and prognostic markers across various cancer types, playing a crucial role in the advancement of precision and personalized oncology treatment.

In this study, we have delineated a subpopulation of tumour cells associated with the progression of LUAD and developed a predictive model for LUAD based on the key identification genes of this subpopulation. Initially, through a multi‐faceted analysis of scRNA‐seq data, we established the linkage between Cluster6 and LUAD progression. This was corroborated through various modalities, including the MK signalling pathway, trajectory analysis and differential gene expression studies. Subsequently, from the array of marker genes identified in Cluster6, we selected nine pivotal genes (PSMB1, ENY2, NPTN, RPS16, HYAL3, KLK8, CAMTA1, MAGEH1 and AKR1A1) to construct a prognostic model for LUAD.

The high‐LPAS group exhibited generally poorer prognoses, which could be attributed to the activation of specific signalling pathways, including porin activity, G2/M DNA replication checkpoint, the folding of actin by CCT/TRiC and the positive regulation of telomerase RNA localization to the Cajal body. Moreover, to validate the predictive efficacy of our model, we conducted a comparative analysis against 144 previously published models. Our findings revealed that in the data sets TCGA, GSE13213, GSE29016, GSE30219, GSE31210 and GSE68465, the c‐index of the LPAS surpassed those of models presented in other published articles. This indicates that our model holds substantial promise as a diagnostic tool for prognostication in LUAD. Additionally, we assessed the performance of the LPAS in terms of immune infiltration, enrichment analysis, and response to immunotherapy. All these evaluations further validated the robust predictive capability of the LPAS model. Parallel conclusions were drawn from in vitro experimental validations. Consequently, it is plausible to posit that our model can accurately predict the prognosis of LUAD patients and their responsiveness to immunotherapies.

Many previous studies have discovered the role of PSMB1 in tumour development. Research by Teng and others found that high expression of PSMB1 is associated with poorer prognosis in breast cancer patients.^44^ Similar results have also been found in diseases such as osteosarcoma, thyroid cancer and ovarian cancer.^45^, ^46^, ^47^ In this study, PSMB1 demonstrated high expression in LUAD, indicating a shorter OS period. After knocking down the PSMB1 gene, the proliferation and DNA replication capabilities of LUAD cells were significantly inhibited. Therefore, we believe that PSMB1 plays a very important role in the progression of LUAD.

This study has several limitations. First, the mechanisms by which these model genes influence LUAD progression require further in‐depth research. Second, no further analysis was performed on the compounds obtained from CTRP and PRISM to clarify potential therapeutic drugs. Third, the model requires more in vitro testing and clinical validation.

We identified a specific tumour cluster associated with the progression of LUAD and successfully developed a high‐performing LPAS. We also observed differences in CNVs and biological functions between high‐ and low‐LPAS groups. In addition, we studied potential mechanisms leading to poor prognosis in the high‐LPAS group and identified potential therapeutic targets, providing new insights for precision treatment.

## AUTHOR CONTRIBUTIONS

**Pengpeng Zhang:** Conceptualization (equal); methodology (equal); supervision (equal). **Zijun Yang:** Data curation (equal); validation (equal). **Zuo Liu:** Formal analysis (equal); visualization (equal); writing – original draft (equal). **Ge Zhang:** Investigation (equal); validation (equal). **Lianmin Zhang:** Funding acquisition (equal); visualization (equal); writing – original draft (equal). **Zhenfa Zhang:** Funding acquisition (equal); validation (equal). **Jun Fan:** Methodology (equal); visualization (equal).

## FUNDING INFORMATION

This work was supported by the Tianjin Natural Science Foundation (grant/award number 21JCYBJC01020).

## CONFLICT OF INTEREST STATEMENT

The authors hereby declared that the research was carried out without any potential conflict of interest arising from commercial or financial relationships.

## DECLARATIONS

The human experiments conducted in this study received ethical approval from the Ethics Committee of the First Affiliated Hospital of Nanjing Medical University. Informed consent was obtained from all participants prior to their involvement. The study adhered to the principles outlined in the Declaration of Helsinki and followed ethical guidelines.

## Supporting information


Table S1.

## Data Availability

The data sets analysed in the current study are available in the TCGA repository (http://cancergenome.nih.gov/) and GEO (https://www.ncbi.nlm.nih.gov/geo/).
